# Effects of Polystyrene Microplastic Exposure on Liver Cell Damage, Oxidative Stress, and Gene Expression in Juvenile Crucian Carp (*Carassius auratus*)

**DOI:** 10.3390/toxics13010053

**Published:** 2025-01-12

**Authors:** Xiangtong Li, Yuequn Huang, Wenrong Li, Chaoyang Deng, Weiyuan Cao, Yi Yao

**Affiliations:** 1 Guangxi Key Laboratory of Theory and Technology for Environmental Pollution Control, Guilin University of Technology, Guilin 541006, China; 19831020251@163.com (X.L.); 2001012@glut.edu.cn (Y.H.); liwenrong2024@126.com (W.L.); dcy1257276184@163.com (C.D.); 2 Engineering Research Center of Watershed Protection and Green Development, Guilin University of Technology, Guilin 541006, China; 3 Guangxi Engineering Research Center of Comprehensive Treatment for Agricultural Non-Point Source Pollution, Guilin University of Technology, Guilin 541006, China; 4 Modern Industry College of Ecology and Environmental Protection, Guilin University of Technology, Guilin 541006, China; 5 Collaborative Innovation Center for Water Pollution Control and Water Safety in Karst Area, Guilin University of Technology, Guilin 541006, China

**Keywords:** polystyrene microplastics, variant liver oxidative damage, antioxidant enzymes, antioxidant-related genes

## Abstract

A considerable quantity of microplastic debris exists in the environment and the toxicity of these materials has a notable impact on aquatic ecosystems. In this paper, 50–500 µm polystyrene microplastics (exposure concentrations were 200 µg/L, 800 µg/L, and 3200 µg/L concentrations) were selected to study the effects of polystyrene microplastics (PS-MPs) on cell morphology, detoxification enzyme activity, and mRNA expression in the liver tissues of crucian carp juveniles. The results demonstrated that: (1) Different concentrations of PS-MPs cause varying degrees of pathological and oxidative damage to liver tissue cells of crucian carp. The higher the concentration of microplastics, the lower the antioxidant enzyme (CAT, GST, SOD) activity and the greater the tissue cell damage. These results demonstrate a typical dose–effect relationship. (2) Principal component analysis and Spearman’s correlation analysis demonstrated that four components, namely glutathione S-transferase (GST) and its related genes (*GSTpi*, *GSTα*), along with catalase (CAT), contributed the most to the observed outcome. These four components demonstrated a relatively high level of responsiveness to PS-MP exposure and can be employed as ecotoxicological indicators of microplastics. (3) This experiment evaluated five genes in three treatments, which found that PS-MPs had different effects on gene expression in the liver and the tested genes were involved in different response pathways associated with virulence. In this study, the toxicity of PS-MPs to crucian carp was determined at the cellular, protein, and mRNA expression levels, and combined with principal component analysis and correlation analysis to identify response sensitivity indicators that provide a scientific basis for ecological risk assessment and the safe use of microplastics.

## 1. Introduction

Microplastics (MPs) are generally defined as tiny plastic fragments or particles less than 5 mm in diameter [1]. Recently, the problem of MP contamination in water systems has become increasingly crucial and several scientists have detected MPs in aquatic environments [2,3] and organisms [4,5] worldwide. The effects and dangers of MPs in fish have caused widespread concern worldwide. Jabeen [6], Zhao Jia [7], and Zhang [8] conducted studies on goldfish, zebrafish, and swordtails and found that MPs affect fish tissues, such as the stomach, liver, and intestines. The liver, being the primary detoxification organ in the body, is particularly vulnerable to impairment and toxicity from MPs, making it a main target for these contaminants, entering the liver tissue through circulation [9], inducing inflammation in liver tissue cells, and interfering with the normal metabolism of the liver [10], resulting in varying degrees of pathology.

Carassius auratus is a freshwater fish species that is highly sensitive to various environmental pollutants. It is commonly used as a representative of aquatic ecological groups and a test organism for toxicological analyses. Furthermore, it is considered a promising species for assessing plastic pollution [11,12]. As the major detoxifying organ in crucian carp, damage to or pathological changes in the liver can lead to metabolic disorders, immune dysfunction, and even death [13]. The study of the effects of MPs on juvenile crucian carp liver is highly representative.

The Ingestion of MPs Induces the production of reactive oxygen radicals (ROS) and oxidative stress in fish [14]. In fish, an imbalance between ROS production and elimination results in DNA damage, protein oxidation, lipid peroxidation, and enzymes [15,16]. The liver is an important antioxidant defense organ in fish and contains various antioxidant enzymes, including superoxide dismutase (SOD), glutathione S-transferase (GST), and catalase (CAT), which are essential for the body to scavenge ROS [17]. The antioxidant defense system exerts a regulatory influence on ROS metabolism, thereby preventing the development of pathological changes. Malondialdehyde (MDA) is a by-product of lipid peroxidation caused by ROS. Therefore, MDA levels can serve as an indicator of the overall lipid peroxidation within an organism’s cells [18]. Furthermore, it is a rapid, sensitive, and effective method for determining the degree of stress in organismal tissues based on changes in the mRNA expression of antioxidant defense-related genes. Cytochrome P450 enzyme 1A is a phase I detoxification enzyme [19]. The expression of the gene encoding cytochrome P450 enzyme 1A (*CYP1A*) was found to undergo significant changes in response to external pollution stress. GST is a detoxification enzyme that acts in conjunction with CAT during phase II detoxification. The expression of GST-related genes, including *GSTpi* and *GSTα*, can serve as a highly sensitive indicator of the antioxidant defense system of an organism. Vitellogenin-like (Vtg) is the precursor of vitellogenin, a large molecular weight phosphoglycoprotein synthesized by the liver in response to the stimulation of yolk-forming estrogen. The gene *Vtg1*, which is associated with this process, is also subject to regulation by external stressors [20]. The *ChgH* gene, as an important eggshell precursor protein gene, is also commonly used as an indicator of stress in organisms under pollution stress [21].

At present, research on the impact of MPs on fish is somewhat fragmented. Many studies merely describe the physiological toxicity, molecular toxicity, or oxidative stress of various fish tissues. However, this paper combines the three aspects of cells, proteins, and molecules in tissues to conduct an in-depth study on the impact of MPs on the livers of fish. In this paper, we used polystyrene microplastics (PS-MPs) as an exposure source to realistically reflect the effects of MPs on the liver of fish in the natural environment by simulating the liver response of fish after ingestion of MPs. Through a 32 d exposure experiment, we analyzed the effects of MPs on the cellular morphology and immune function of the liver tissue of crucian carp larvae, and investigated the mechanism of the liver response to microplastics in crucian carp juveniles, as well as the intrinsic linkage of the physiological activities of the liver under PS-MPs contamination at the cellular, protein, and molecular levels.

## 2. Materials and Methods

### 2.1. Instruments and Reagents

The instruments used for sample processing were: JXFSTPRP-48 Automatic Sample Rapid Grinder (Shanghai Jingxin Industrial Development Co., Ltd., Shanghai, China), Allegra 64R Centrifuge Freezing Centrifuge (Beckman coulter, Indianapolis, IN, USA), CM1850 Leica Frozen Slicer (Leica Biosystems, Deer Park, IL, USA), and thermostatic water bath (35–90 °C). Fish tissues were observed using a Pantherai-type biomicroscope (McAuldie Industrial Group, Ltd., Xiamen, China). Samples were analyzed using a Q5000 Ultra-micro UV Spectrophotometer (Quawell Technology, Sunnyvale, CA, USA), Quant Studio3 Real-Time Fluorescence PCR (Applied Biosystems, Waltham, MA, USA), and a 2027 Thermal cycler Amplifier (Applied Biosystems).

The microplastics utilized in the experiment were obtained from polystyrene microplastic foam lunch boxes. Following the crushing of the meal box through the foam box grinder, the experimental microplastics were selected through a 50–500 µm steel screen. The microplastics were found to be present in the form of strips and granules. The rest of the chemical reagents were analytically pure.

### 2.2. Test Organism

Crucian carp juveniles were purchased from the Huizhou Ecological Farm, Guangdong Province, China. Prior to the experiment, crucian carp juveniles were domesticated in a glass tank for 14 d. The tank was provided with 24 h of uninterrupted aeration and maintained at a temperature of 23 ± 1 °C. The light cycle was set at 14 h of light and 10 h of dark. It is recommended that dehulled *Artemiasaline* eggs be fed once a day, and that residual bait and excreta be removed in a timely manner. The mortality rate of less than five percent during the domestication process met the criteria for experimental fish. Baiting was terminated one day prior to the commencement of the experiment, and randomly selected healthy and uniformly sized crucian carp juveniles for the experiment, with an average body length of (3.03 ± 0.22) cm and an average body weight of (0.80 ± 0.26) g. Oxygenation was maintained throughout the duration of the experiment.

### 2.3. Experimental Methods

#### 2.3.1. Poisoning Method

We referred to previous investigations in the Beibu Gulf [22] and exposure concentrations commonly used in toxicity testing of microplastics in aquatic environments [23]. Three concentration gradients were established for the low-concentration group (L) (200 μg/L), medium-concentration group (M) (800 μg/L), and high-concentration group (H) (3200 μg/L), with a blank group (CK) (without PS-MPs) designated as the control. Four parallel experimental and control groups were established. Four parallel groups were set up for the experimental and blank groups, with 6 L of experimental solution in a glass fish tank (30 cm × 18 cm × 20 cm) with 12 fish per tank. The experimental water was dechlorinated via aeration for 24 h. The pH was recorded as 7.0–7.3, the temperature was 23 ± 1 °C, the dissolved oxygen was ≥5.0 mg·L^−1^, the water was completely changed at 16:00 each day, and the food was administered at 9:00 each day.

#### 2.3.2. Collection of Tissue Samples

Following a 32-d exposure period, crucian carp juveniles were anesthetized with MS-222. Body length and weight were measured, and the experimental fish were dissected. Liver tissues were removed and rinsed in 0.9% saline at 4 °C to remove blood. They were then weighed by wiping dry with filter paper, numbered, and placed in a −80 °C refrigerator for freezing and storage.

#### 2.3.3. Methods of Histopathological Analysis

Following the completion of the exposure experiments, seven carp were selected from each concentration group, and their liver tissues were transferred to sterile centrifuge tubes containing 10% formaldehyde to preserve the samples at room temperature. Cot embedding agent was used for embedding and freezing, and the slices were sectioned to a thickness of 2 mm using a Leica frozen slicer. Hematoxylin and eosin staining was performed, and the slices were subsequently sealed with a neutral resin. Following drying, the cells were observed under a biomicroscope, and the sectioned tissues were photographed using the Motic software (Motic Image Plus 2.0).

#### 2.3.4. Determination of Indices Related to Antioxidant Enzyme Activity

Following exposure to each concentration, juvenile crucian carp liver tissue was collected and pre-cooled saline was added according to the sample mass at a ratio of 1:9 between the sample and saline (medicine). This was followed by the addition of sterilized zirconia beads. Tissue homogenate was ground in a fully automated sample rapid grinder, followed by centrifugation in a cryo-centrifuge at 3000 rpm for 10 min at 4 °C, and the supernatant was collected and processed into a 1% tissue homogenate for antioxidant enzyme activity assay. CAT, SOD, GST, MDA, and total protein (TP) were determined using kits from Nanjing Jiancheng Bioengineering Institute (Nanjing, China) and the specific operation was performed according to the instructions of the kits.

#### 2.3.5. Analysis of mRNA Expression of Antioxidant-Related Genes

(1) Total RNA extraction and cDNA synthesis

Total RNA was extracted from the liver using the RNAiso Plus kit (TaKaRa, San Jose, CA, USA) according to the TRIzol method. RNA was synthesized into first-strand cDNA for fluorescence quantitative PCR experiments using the PrimeScript RT Reagent Kit with a gDNA Eraser kit (TaKaRa).

(2) Fluorescence quantitative PCR

qPCR-specific primers were designed according to the juvenile crucian carp gene sequence (Table 1) and real-time fluorescence quantitative PCR experiments were performed using a fluorescence quantitative PCR instrument. qPCR experiments were performed using the SYBR Premix Ex TaqTM (TaKaRa) kit, and the total reaction system was 10 μL of SYBR Premix MixTaq (2×), 1 μL each of upstream and downstream primers (10 μmol-L^−1^), 2 μL of cDNA, and 6 μL of dH_2_O. The reaction procedure was the following: permutability 95 °C, 5 min; 40 cycles 95 °C, 20 s; 60 °C, 20 s; 72 °C, 30 s. β-actin was selected as the housekeeping gene and liver target genes included eggshell precursor protein (*ChgH*), vitellogenin (*Vtg1*), CYP1A, and transferases (*GSTpi*, *GSTα*).

### 2.4. Data Processing Methods

The 2^−∆∆CT^ method was used to analyze the relative gene mRNA expression, and all experimental data were expressed as (mean ± standard error). The data obtained were analyzed and processed using Origin 2021 and IBM SPSS 27 software. “*” indicates a significant difference (*p* < 0.05) and “**” indicates a highly significant difference (*p* < 0.01).

## 3. Results

### 3.1. Histological Analysis of PS-MP Stress on Liver Cell Destruction in Juvenile Crucian Carp

**Figure 1:**
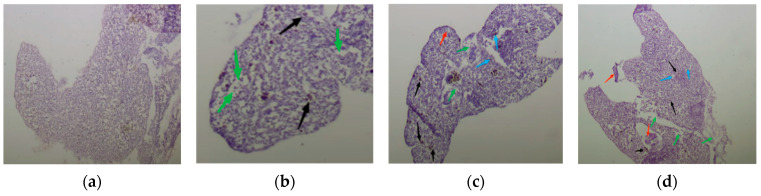
Cellular morphology of liver tissue of juvenile crucian carp after 32 d of stress by PS-MPs: (**a**) blank control group; (**b**) low-concentration group; (**c**) medium-concentration group; (**d**) high-concentration group (Black arrows indicate cell congestion, red arrows indicate vacuolated cells, green arrows indicate off-center nuclei, and blue arrows indicate enlarged necrotic cells, Picture magnification is 40×).

The liver is an important detoxification organ in animals and MPs induce oxidative stress in the body, which disrupts lipid metabolism in the liver tissue and the stability of liver cells, causing cellular damage [24]. Figure 1 shows the morphology of juvenile crucian carp liver tissue cells at low, medium, and high levels of microplastic exposure and a blank control.

As illustrated in Figure 1, the livers of the control group juvenile crucian carp exhibited a normal tissue structure. Hepatocyte nuclei were larger and located in the center of the cells, with all cell row types appearing neat and close. Additionally, the structure of the glandular vesicles was normal without any evidence of pathological abnormalities (Figure 1a). The liver tissue structure of juvenile crucian carp in the low-concentration group was essentially normal and exhibited only minor abnormalities. A subset of cells exhibited off-center nuclei and cell enlargement. (Figure 1b). The liver tissue structure of juvenile crucian carp in the medium-concentration group exhibited mild abnormalities, including hyperemia in liver tissue cells, a small portion of liver cells that had enlarged and undergone necrosis, and nuclei of liver cells that deviated from the center (Figure 1c). The liver tissue structure of juvenile crucian carp in the high-concentration group exhibited severe abnormalities, characterized by a high degree of cellular hyperemia in the liver tissue cells and a greater degree of hepatocyte enlargement and necrosis. Additionally, the nuclei of the hepatocytes deviated from the center of the cells, accompanied by inflammation and severe cellular vacuolation (Figure 1d). These results demonstrated that the liver tissue cells of juvenile crucian carp exhibited lesions and necrosis because of exposure to PS-MPs. Furthermore, the degree of liver tissue cell destruction increased proportionally with the concentration of PS-MPs.

### 3.2. Changes in Antioxidant Enzyme Activities in Liver Tissues of Juvenile Crucian Carp After 32 d of Exposure to PS-MPs

**Figure 2:**
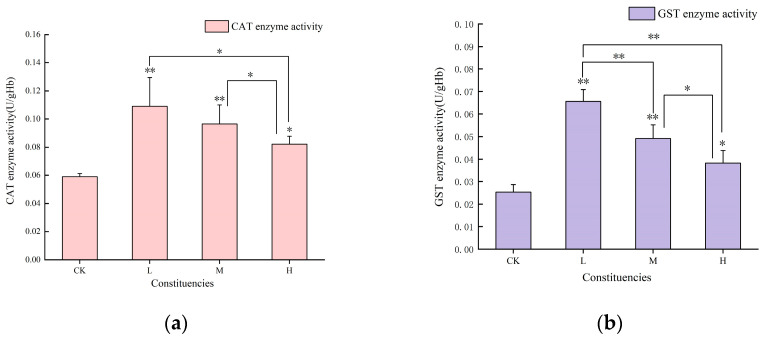

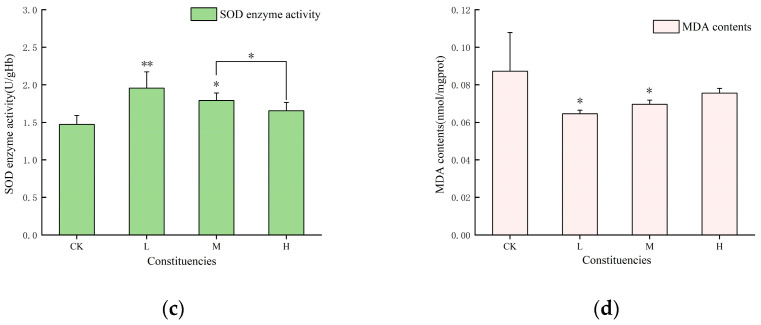
Changes in antioxidant enzymes in liver tissues of juvenile crucian carp after 32 d of exposure to PS-MPs: (**a**) superoxide dismutase (SOD) activity, (**b**) glutathione S-transferase (GST) activity, (**c**) catalase (CAT) activity, (**d**) malondialdehyde (MDA) content. “*” indicates a significant difference (*p* < 0.05) and “**” indicates a highly significant difference (*p* < 0.01).

The liver detoxification enzyme activities of the three experimental groups in this thesis were higher than those of the blank control group, which was complementary to the literature description. With the increase in microplastic concentration, the activities of liver detoxification enzymes were gradually weakened, which showed that the activities of CAT, GST and SOD showed a “stepwise decrease”, while MDA showed a “stepwise increase”, and the activities of antioxidant enzymes in the liver tissues showed a dose- effect relationship in liver tissue. (Figure 2). The activity of CAT, GST, and SOD was elevated in the exposed group compared to that in the blank group, whereas the MDA content was diminished in the former in contrast to the latter. In comparison, CAT activity was markedly elevated (*p* < 0.01) in the low- and medium-concentration groups, exhibiting 1.75- and 1.64-fold increases, respectively, relative to the control group. Furthermore, the high-concentration group demonstrated a statistically significant increase (*p* < 0.05), exhibiting a 1.40-fold increase relative to that in the control group (Figure 2a). The low- and medium-concentration groups exhibited markedly elevated (*p* < 0.01) levels of GST activity, with the former displaying a 2.59-fold increase and the latter displaying a 1.94-fold increase relative to the control group. The high-concentration group demonstrated a significant (*p* < 0.05) increase in GST viability, with the latter exhibiting a 1.51-fold increase compared to the control group (Figure 2b). The change in SOD activity was highly significant (*p* < 0.01) in the low-concentration group, which exhibited a 1.33-fold increase relative to the control group. This was also significant (*p* < 0.05) in the medium-concentration group, which demonstrated a 1.22-fold increase relative to the control group. However, no significant difference in SOD activity was observed at high concentrations, relative to the control group (Figure 2c). The MDA content was significantly reduced (*p* < 0.05) in the low- and medium-concentration groups, with reductions of 26% and 20%, respectively. (Figure 2d).

### 3.3. Changes in mRNA Expression of Antioxidant-Related Genes in Juvenile Crucian Carp Liver Tissues After 32 d Exposure to PS-MPs

**Figure 3:**
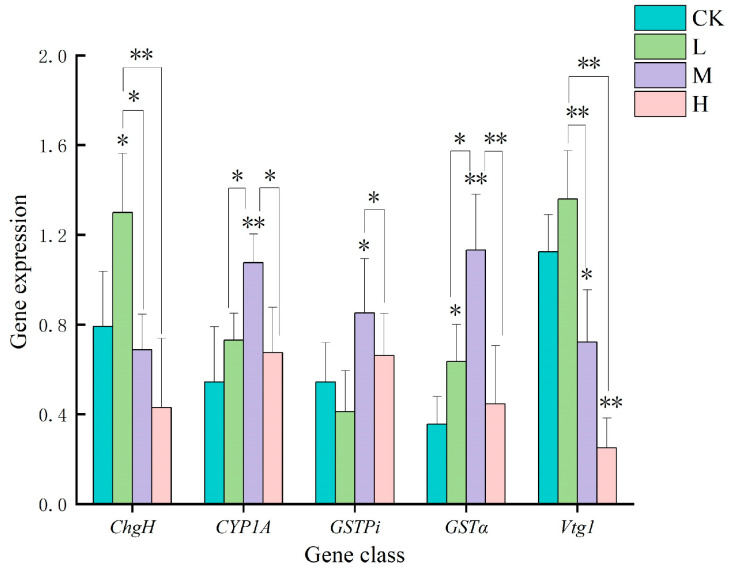
Changes in mRNA expression of antioxidant-related genes in liver tissues of juvenile crucian carp after 32 d of PS-MP exposure. “*” indicates a significant difference (*p* < 0.05) and “**” indicates a highly significant difference (*p* < 0.01).

After 32 d of exposure to PS-MPs, the mRNA expression of *ChgH* in juvenile crucian carp liver tissues gradually decreased with increasing exposure concentration. The *ChgH* gene exhibited a significant increase in expression in the low-concentration group (*p* < 0.05), with a 1.64-fold increase compared with the control group. Conversely, the *ChgH* gene demonstrated a decline in expression in the middle- and high-concentration groups relative to the blank group (Figure 3). The mRNA expression of the *CYP1A* gene in the experimental group was elevated compared to that in the blank group. Furthermore, a pattern emerged in which the expression initially increased and subsequently decreased with an increase in the exposure concentration. The *CYP1A* gene exhibited a significant increase in expression in the middle-concentration group (*p* < 0.01), which was 1.98 times higher than that observed in the control group. No significant difference was evident between the low- and middle-concentration groups and the blank group. The expression of *GSTpi* gene mRNA demonstrated a tendency to increase and subsequently decline with increasing exposure concentrations. The expression of the *GSTpi* gene was found to be significantly upregulated in the intermediate concentration group (*p* < 0.05), exhibiting a 1.57-fold increase relative to the control group. Conversely, the expression decreased in the low-concentration group compared to that in the blank group and increased in the high-concentration group. The mRNA expression of the *GSTα* gene in the experimental group was observed to be elevated in comparison to that of the blank group. Furthermore, a pattern emerged whereby the expression initially increased and then decreased with an increase in the exposure concentration. Among the observed variables, gene expression exhibited a statistically significant increase in the low-concentration group (*p* < 0.05), with a 1.79-fold increase relative to the control group. Furthermore, a statistically significant increase was observed in the medium-concentration group (*p* < 0.01), with a 3.18-fold increase relative to that in the control group. Conversely, the high-concentration group demonstrated a relatively modest increase compared with that in the experimental group. The mRNA expression of *Vtg1* gene showed a gradual decrease with the increase in exposure concentration, in which the expression of the low-concentration group slightly increased compared to that in the blank group, whereas the mRNA expression of *Vtg1* gene in the middle-concentration group significantly decreased by 36% compared to that in the blank group (*p* < 0.05), and the mRNA expression of *Vtg1* gene in the high-concentration group significantly decreased by 78% compared to that in the blank group (*p* < 0.01).

### 3.4. Principal Component Analysis and Correlation Analysis

**Figure 4:**
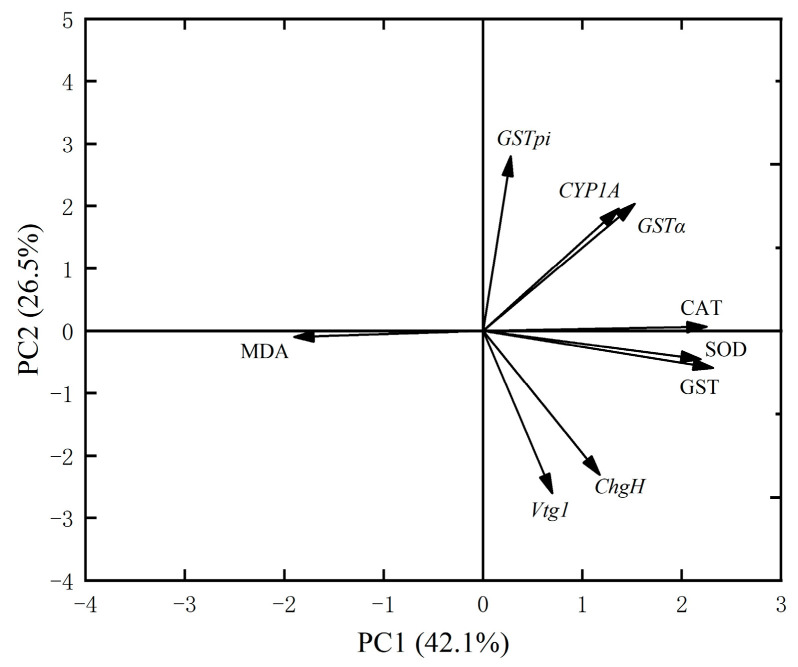
Principal component analysis of antioxidant enzymes and related genes in liver tissue of juvenile crucian carp after 32 d of PS-MP exposure.

According to the PCA, the cumulative contribution of the first principal component (PC1) and the second principal component (PC2) in the liver was 68.6% (Figure 4); in the PC1 eigenvector, the *GSTpi* gene and *GSTα* gene contributed the most and in the PC2 eigenvector, the GST enzyme and CAT enzyme contributed the most, which indicated that juvenile crucian carp liver tissues *GSTpi* gene, *GSTα* gene, GST enzyme, and CAT enzyme were most affected by MPs.

**Figure 5:**
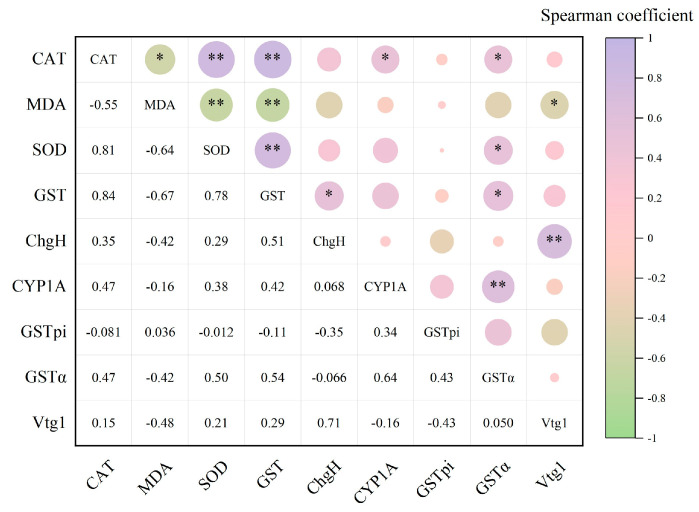
Heatmap of correlation analysis of antioxidant enzymes and related genes in liver tissues of juvenile crucian carp after 32 d of PS-MP exposure. “*” indicates a significant difference (*p* < 0.05) and “**” indicates a highly significant difference (*p* < 0.01).

According to Spearman’s correlation analysis, in liver tissues (Figure 5), the CAT enzyme showed a highly significant positive correlation with the SOD enzyme (*ρ* = 0.81, *p* < 0.01) and the GST enzyme (*ρ* = 0.84, *p* < 0.01), respectively, and a significant positive correlation with the CYP1A gene (*ρ* = 0.47, *p* < 0.05) and GSTα gene (*ρ* = 0.47, *p* < 0.05) correlation, and significant negative correlation with MDA content (*ρ* = −0.55, *p* < 0.05). MDA content showed highly significant negative correlation with the SOD enzyme (*ρ* = −0.64, *p* < 0.01) and GST enzyme (*ρ* = −0.67, *p* < 0.01) and significant negative correlation with the *Vtg1* gene (*ρ* = −0.48, *p* < 0.05), respectively. The SOD enzyme showed a highly significant positive correlation with the GST enzyme (*ρ* = 0.78, *p* < 0.01) and a significant positive correlation with the *GSTα* gene (*ρ* = 0.50, *p* < 0.05). GST enzymes were significantly and positively correlated with the *ChgH* gene (*ρ* = 0.51, *p* < 0.05) and *GSTα* gene (*ρ* = 0.54, *p* < 0.05), respectively, whereas the *ChgH* gene showed a highly significant positive correlation with the *Vtg1* gene (*ρ* = 0.71, *p* < 0.01); the *CYP1A* gene showed a highly significant positive correlation with the *GSTα* gene (*ρ* = 0.64, *p* < 0.01).

In conclusion, principal component analysis and Spearman’s correlation analyses revealed a strong correlation between antioxidant enzymes in juvenile crucian carp liver tissues. Furthermore, the activities of antioxidant enzymes and the mRNA expression of related genes were found to be positively correlated. In juvenile crucian carp liver tissues, the *GSTpi* gene, the *GSTα* gene, the GST enzyme, and the CAT enzyme exhibited greater effects after 32 d of exposure to PS-MPs. These effects can be used as indicators of the ecotoxicological impact of microplastics.

### 3.5. Relationship Between Antioxidant Enzyme Activities and Related Genes in Juvenile Crucian Carp After 32 d of MP Exposure

#### 3.5.1. Interrelationship Between GST Enzymes and Genes and PS-MP Exposure 32 d in Juvenile Crucian Carp Liver Tissue

**Figure 6:**
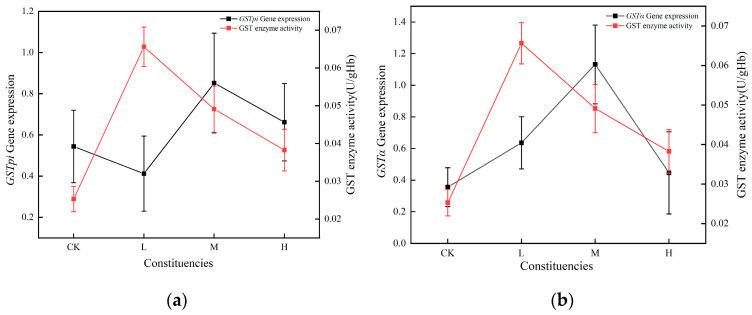
Relationship between antioxidant enzymes and corresponding genes in juvenile crucian carp liver tissues after 32 d of PS-MP exposure: (**a**) Relationship between GST enzyme activity and *GSTpi* gene expression; (**b**) Relationship between GST enzyme activity and *GSTα* gene expression.

In juvenile crucian carp liver tissues, the alterations in GST activity, with the exception of the low concentration, were in accordance with the trends observed in mRNA expression of *GSTα* and *GSTpi* genes. The highest gene expression was observed at the middle concentrations, whereas a decline was observed at high concentrations. The GST enzyme was significantly positively correlated with the *GSTα* gene in liver tissues (Figure 6b) and not significantly correlated with the *GSTpi* gene (Figure 6a).

#### 3.5.2. Response of Antioxidant-Related Genes in Juvenile Crucian Carp Liver Tissue to 32 d Exposure to PS-MPs

**Figure 7:**
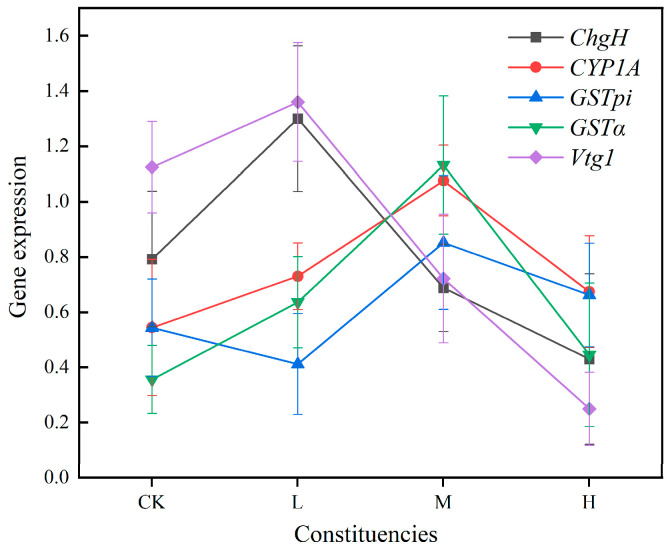
Trends of mRNA expression of antioxidant-related genes in liver tissues of juvenile crucian carp after 32 d of PS-MP exposure.

In juvenile crucian carp liver tissues (Figure 7), the trends of *CYP1A* and *GSTα* gene expression changes in different concentration groups were consistent, exhibiting a pattern of initial increase and subsequent decrease in expression with rising exposure concentration. Additionally, the expression of *GSTpi* demonstrated an initial increase and subsequent decrease at elevated concentrations. However, expression in the low-concentration group was lower than that in the blank group. The expression of *ChgH* and *Vtg1* in the various concentration groups exhibited a comparable trend, yet the expression tended to decline with increasing exposure concentration. The expression in the middle- and high-concentration groups was also lower than that in the blank control group.

## 4. Discussion

### 4.1. Effects of 32 d Exposure of PS-MPs on Liver Tissue Cells of Juvenile Crucian Carp

The introduction of PS-MPs into the circulation of an organism affects the stability of tissue and cell morphology, resulting in varying degrees of damage to cell membranes. This, in turn, leads to the lipid peroxidation of cell membranes and the induction of a range of pathological phenomena in cells [25]. The results of this study demonstrated that the liver tissue cells of juvenile crucian carp exhibited varying degrees of damage when exposed to PS-MPs. The extent of this damage increased with increasing concentrations of PS-MPs. The liver tissue cells were observed to maintain a normal physiological state without significant lesions when exposed to 200 μg/L PS-MPs. Upon elevating PS-MPs to 800 μg/L, disturbances in liver tissue cell morphology were observed, with a small proportion of cells exhibiting hemorrhagic and enlarged characteristics. The data indicated no evidence of excess cell death in the liver cells at either concentration. This finding is consistent with the results of previous studies that demonstrated that low concentrations do not cause significant cell lethality [26]. Upon reaching 3200 μg/L, PS-MPs were introduced to juvenile crucian carp liver cells, resulting in notable alterations to the cellular membranes and a considerable reduction in the number of tight junctions. The loss of cell–cell contact served as the initial indicator of morphological alterations in tissue cells [27], which ultimately resulted in the destabilization of liver cell tissues subjected to a 3200 μg/L concentration of PS-MPs. This concentration led to the manifestation of notable pathological phenomena, including necrosis and inflammation, and caused severe tissue damage. Moreover, high concentrations of MPs can also affect the liver insulin signaling pathway, influence the normal distribution and transport of calcium ions in stem cells, and cause the stability of lysosomal membranes to deteriorate or even rupture. All these reasons may lead to damage to liver tissues.

### 4.2. Effects of 32 d Exposure to PS-MPs on the Hepatic Antioxidant System of Juvenile Crucian Carp

MPs can induce an increase in ROS, resulting in changes in the fish liver tissue and oxidative stress [28]. Although the liver has its own antioxidant defense system, a large amount of ROS induced by high concentrations of MPs may exceed the scavenging capacity of the antioxidant system. As a result, antioxidant substances are consumed in large quantities and gradually depleted, leading to a decrease in the activity of detoxification enzymes. After 32 d of exposure to PS-MPs, the liver tissue antioxidant enzyme activities and related genes showed concentration-specific changes in the three concentration-treated groups of juvenile crucian carp. MPs also induce the expression of antioxidant genes [29,30]. The mRNA expression of the *CYP1A* and *GSTα* genes was observed to be upregulated in this experiment, whereas the expression of the *GSTpi* gene was found to be downregulated in the low-concentration group and subsequently upregulated in the middle- and high-concentration groups. This suggests that MPs have a unique regulatory effect on gene expression. The CAT, GST, and SOD enzyme activities in the experimental group were notably higher than those in the blank group. In the low-concentration group, considerable increases in CAT, GST, and SOD enzyme activities were observed. Conversely, as the concentration of PS-MPs increased, enzyme activity gradually declined. This finding aligns with the results of the previous literature review [24,31].

Concomitantly, CAT, GST, and SOD enzyme activities exhibited similar patterns of sequential and gradual declines with increasing concentrations. This gradual decrease in enzyme activity may be due to the excessive accumulation of ROS produced by high concentrations of MPs, inducing changes in the synthesis, inactivation, or transcriptional expression of relevant enzymes [32]. CAT enzyme showed highly significant positive correlation with the SOD enzyme (*ρ* = 0.81, *p* < 0.01) and GST enzyme (*ρ* = 0.84, *p* < 0.01), respectively, and the SOD enzyme showed highly significant positive correlation with the GST enzyme (*ρ* = 0.78, *p* < 0.01). This suggests a synergistic effect of CAT, GST, and SOD under MP exposure.

MDA, a product of lipid peroxidation, gradually increased with increasing concentrations of PS-MPs; however, was lower in the different treatment groups than in the blank group. Meanwhile, MDA content showed highly significant negative correlation with the SOD enzyme (*ρ* = −0.64, *p* < 0.01) and GST enzyme (*ρ* = −0.67, *p* < 0.01), respectively. This indicates that the increased concentration of MPs led to a decrease in the activity of detoxification enzymes in the fish, resulting in an increase in the ROS content. The excess ROS caused an increase in cellular lipid peroxidation, which is consistent with the changes in the MDA content.

### 4.3. Interrelationship of Antioxidant Enzymes and Genes in the Liver of Juvenile Crucian Carp to PS-MPs for 32 d

After entering the fish organism, MPs form a physical barrier through adsorption, affecting the asynchrony of gene expression and enzyme activity changes. At the same time, MPs can trigger oxidative stress, and gene expression and antioxidant enzyme activities are affected by ROS to change, showing the incoordination between enzyme activities and gene expression. In addition, the inflammatory response triggered by MPs releases inflammatory factors and regulates the expression of related genes, but the complexity of inflammation and the pleiotropic effect of inflammatory factors on enzyme activity also contribute to the irregularity between gene expression and enzyme activity (Table 2).

After 32 d of exposure to PS-MPs, the trends of the GST enzyme, *GSTα* gene, and *GSTpi* gene changes in juvenile crucian carp liver were the same in the medium- and high- concentration groups. Among the three treatment groups, the GST enzyme showed a significant positive correlation with the *GSTα* gene (*ρ* = 0.54, *p* < 0.05) and a non-significant correlation with the *GSTpi* gene, and the expression of mRNAs of genes related to the antioxidant system of juvenile crucian carp liver was more sensitive to the exposure of 32 d of MPs. After the exposure of fish to MPs, the gene response was more sensitive than the enzyme activity response, which may be related to multiple regulatory factors in the translation of genes into proteins [33]; thus, the enzyme response lagged behind the mRNA expression of the relevant genes. The expression level of mRNA has a regulatory effect on antioxidant enzymes, and the response of mRNA expression is faster and more sensitive than that of enzyme activity. Therefore, to some extent, changes in the expression level of mRNA can reflect the toxicological impacts on organisms more quickly.

**Table 2:** Comparison of phenomena.

Phenomena Observed in This Paper	Phenomena Observed in References
GST enzyme activity and *GSTα* genes were elevated in the low-concentration group, while *GSTpi* genes were reduced in the low-concentration group.	Larimichthys crocea showed a significant increase in GST activity and *GSTα* gene and a decrease in the expression of some antioxidant-related genes under low external stress [34,35].
Antioxidant enzyme activity gradually decreased with increasing microplastic concentration, while *CYP1A* and *GSTα* genes first increased and then decreased.	The GST enzyme activity of yellow catfish exposed to external contamination gradually decreased with the increase in contamination concentration, while the expression of *CYP1A* gene first increased and then decreased [36,37].
GST enzyme activity was highest in the low-concentration group, while *GSTpi* gene and *GSTα* gene expression was highest in the medium-concentration group.	The activity of antioxidant enzymes decreases after reaching the pollution threshold, and the expression of some antioxidant-related genes increases and then decreases [33].

### 4.4. Effects of 32 d Exposure to PS-MPs on the Expression of Antioxidant Genes in the Liver of Juvenile Crucian Carp

PS-MPs induced a large amount of ROS generation and oxidative stress after entering juvenile crucian carp and further stimulated the transcriptional expression of genes related to the antioxidant defense system to eliminate the effects of ROS on the organism [38]. The detoxification of cells in response to external stress includes Phases I, II, and III [39,40]. In the present study, when faced with MP stress, juvenile crucian carp liver *CYP1A* gene and *GSTpi* gene changed with the same trend (*ρ* = 0.64, *p* < 0.01) in a highly significant positive correlation, and this situation suggests that the *CYP1A* gene, as a phase I detoxification enzyme gene, has a certain regulatory effect on phase II detoxification enzyme gene *GSTpi*. Conversely, the *GSTα* gene, which is also a phase II detoxification enzyme gene, exhibits reduced expression at low concentrations, a phenomenon that differs from that observed with the *CYP1A* gene. Furthermore, the trend of change in the middle- and high-concentration groups was similar, indicating that phase I detoxification enzyme genes may be influenced by alternative regulatory pathways in the context of phase II detoxification enzyme genes. As the concentration of MPs increased, all three detoxification enzyme genes demonstrated an initial elevation followed by a decline. This observation indicates that elevated concentrations of MPs resulted in a gradual reduction in the liver detoxification capacity of juvenile crucian carp, which may be associated with the damage incurred by liver tissue cells and the liver detoxification capacity reaching a critical threshold.

The current study also revealed that *ChgH* and *Vtg1* gene expression trends were consistent, and their expression decreased with increasing PS-MP concentrations, showing a dose-dependent relationship. In fish, the liver plays a crucial role in the synthesis of yolk proteogens and ovalbuminogen in fish [41]. The expression of *ChgH* and *Vtg1*, which are associated with these proteins, increased in the low-concentration group and decreased in the middle- and high-concentration groups. This suggests that juvenile crucian carp in the low-concentration group were affected by the MPs, leading to an increase in the synthesis of yolk proteogens and ovalbuminogen in the liver. MPs induce steroid biosynthesis [42] and it is hypothesized that more steroids were synthesized by juvenile crucian carp in the low-concentration group, leading to the formation of estrogen receptor ligand complexes, which in turn increased the responsive binding of *ChgH* and *Vtg1* genes, ultimately leading to an increase in their transcripts, whereas in the intermediate- and high-concentration groups, this synthesis process was inhibited. The finding in the present study that MPs can affect the transcriptional expression of genes other than detoxification enzymes also suggests that the effects of MPs on the organism are multifaceted, and that future research should be approached from multiple perspectives.

## 5. Conclusions and Prospects

The presence of microplastics induced excessive reactive oxygen production. The present study revealed that an increase in the concentration of microplastics affects the activity of liver-related detoxification enzymes and the expression of related genes in juvenile crucian carp. This, in turn, affects the antioxidant defense system of juvenile crucian carp, leading to changes in ROS metabolism. These changes ultimately result in the development of lesions. Based on the above findings, and considering the complexity of the effects of microplastics, a multifaceted approach should be adopted in the future to investigate the effects of microplastics on various aspects of the liver, leading to the following conclusions:

(1) Microplastics induced significant changes in the expression of Vtg and Chg-related genes and the content of Vtg and Chg may be an important factor affecting cell morphology, which should be investigated in the future;
(2) Owing to the characteristics of microplastics, the surface can easily adsorb the remaining contaminants, and future studies should systematically analyze the effects of microplastics and other contaminants in fish liver tissues in conjunction with microplastics;
(3) Considering the diversity of microplastic species, shapes, and particle sizes in nature, future studies can be combined with the field environment to design experiments to investigate the state of fish liver tissues when they are subjected to microplastic stress in the field environment.

## Figures and Tables

**Table 1:** Sequences of qPCR primers for target genes.

Gene Name	Upstream Primer Sequence Number	Downstream Primer Sequence Number
*GSTα*	CCCGAGAATATAAAACTCCC	TCAAAAACACTTCCTCAAAC
*Vtg1*	TAGAGCTGGAATGGGAGAGG	TGACACTGTCATCTCTGGAA
*GSTpi*	ATCTACCAGGAATATGAGAC	CGGGCAGCAATCTTATCCAC
*ChgH*	TTGTGGCACCACAATGAAGA	TGGAGGAGGAACAGTGTTGA
*CYP1A*	ATTTCATTCCCAAAGACACCTG	CAAAAACCAACACCTTCTCTCC

## Data Availability

The original contributions presented in the study are included in the article, further inquiries can be directed to the corresponding authors.
